# Optimized scheduling study of user side energy storage in cloud energy storage model

**DOI:** 10.1038/s41598-023-45673-4

**Published:** 2023-11-01

**Authors:** Huidong Wang, Haiyan Yao, Jizhou Zhou, Qiang Guo

**Affiliations:** 1 State Grid Zhejiang Hangzhou Yuhang District Power Supply Company, Hangzhou, 311100 China; 2 Hangzhou Power Equipment Manufacturing Company Limited, Hangzhou, 310000 China

**Keywords:** Energy science and technology, Engineering

## Abstract

With the new round of power system reform, energy storage, as a part of power system frequency regulation and peaking, is an indispensable part of the reform. Among them, user-side small energy storage devices have the advantages of small size, flexible use and convenient application, but present decentralized characteristics in space. Therefore, the optimal allocation of small energy storage resources and the reduction of operating costs are urgent problems to be solved. In this study, the author introduced the concept of cloud energy storage and proposed a system architecture and operational model based on the deployment characteristics of user-side energy storage devices. Additionally, a cluster scheduling matching strategy was designed for small energy storage devices in cloud energy storage mode, utilizing dynamic information of power demand, real-time quotations, and supply at the load side. Subsequently, numerical analysis was conducted to verify that the proposed operational mode and optimal scheduling scheme ensured the maximum absorption of renewable energy, improved the utilization rate of energy storage resources at the user side, and contributed to peak shaving and load leveling in the power grid. The model put forward in this study represents a valuable exploration for new scenarios in energy storage application.

## Introduction

With global climate change posing a major threat to human society, China has taken on the responsibility of being a major power in addressing the problem of excessive carbon emissions and has proposed a vision of a “Carbon-free” future in which “carbon dioxide emissions will strive to peak by 2030, and efforts will be made to achieve carbon neutrality by 2060” ^1^. The key point to achieve double carbon is to promote the implementation of “Two Alternatives”, namely, clean alternative energy production, energy consumption, electrical energy replacement^2^. The generation of new energy power is significantly influenced by climatic factors and exhibits inherent characteristics of randomness and uncertainty, posing substantial challenges to the secure and stable operation of the power system. Energy storage technologies can effectively facilitate peak shaving and valley filling in the power grid, enhance its capacity for accommodating new energy generation, thereby ensuring its safe and stable operation^3,4^. Based on the above background, at present, China's user-side small energy storage devices present decentralized characteristics in space, and it is difficult for the grid to directly coordinate the call, so there is still a need to deeply study commercialization mode of energy storage industry, which is important to achieve the optimal allocation of energy storage resources and helping the grid to adjust the electricity load.

At present, significant progress has been made by scholars in the field of cloud energy storage. Current research primarily focuses on the operational mechanisms, optimization scheduling, economic benefits, and other aspects of user-side energy storage in the cloud energy storage model.

1. Operational mechanism of user-side energy storage in cloud energy storage mode: the operational mechanism of user-side energy storage in cloud energy storage mode determines how to optimize the management, storage, and release of energy storage resources to reduce user costs, enhance sustainability, and maintain grid stability. Some scholars conducted in-depth research on the application of the sharing economy in the energy sector, with a particular focus on virtual power plants, peer-to-peer energy trading, shared energy storage, and microgrid energy sharing cloud^5–7^. They also delved into future research directions and the challenges ahead. Liu Jingkun et al. established an investment and operation decision model for cloud energy storage operators and users^8^. They validated the model's feasibility using actual load profiles and prices of local users in Ireland under both perfect and imperfect scenarios. Li Xiangyu et al. aimed to minimize total investment and operating costs, establishing a long-term collaborative optimization model for cloud energy storage with multiple energy storage systems^9^. Zhou Renjun et al. considered power balance, cloud energy storage system energy storage device limitations, and grid interaction constraints, optimizing cloud energy storage leasing schemes to achieve the goal of minimizing the overall cost for industrial and commercial users^10^. Li Xianshan et al. introduced cloud energy storage into microgrids to provide users with "virtual energy storage" services, building a coordination and optimization model for ecological games among multiple intelligent agents in microgrids with cloud energy storage^11^.
2. Optimization and scheduling of user-side energy storage in cloud energy storage mode: optimizing the scheduling of user-side energy storage in cloud energy storage mode can maximize the availability of user-side energy storage, balance supply and demand, and efficiently utilize energy resources. Riccardo Remo Appino et al. studied the aggregation of user-side energy storage with time-varying power and energy constraints, proposing an aggregation model suitable for cloud energy storage scheduling^12^. Zhang Ning et al. introduced a modeling method based on a cloud platform to aggregate and schedule energy storage resources, optimizing the grid scheduling problem^13^. Ma Yuncong et al. proposed a point-to-point (P2P) trading model in the form of cloud energy storage, incorporating cooperative game theory^14^. They constructed a two-layer P2P two-stage trading optimization model for cloud energy storage operators, communities, and users, achieving fair benefit distribution among multiple parties. Wu Shengjun et al. analyzed the operation mode of existing energy storage systems, establishing a two-level planning model considering the time scale^15^. Lateef Onaadepo Ibrahim et al. conducted research on methods for achieving optimal frequency and power responses^16^. They proposed an adaptive droop control method based on energy storage systems and implemented the proposed control strategy. They demonstrated that the proposed mechanism could reduce user electricity costs and save energy storage resources. Guo Yizong et al. analyzed the energy coordination optimization mechanism of cloud energy storage and microgrids operating jointly, utilizing cloud energy storage coordination scheduling technology to rapidly suppress output power fluctuations^17^.
3. Economic benefits of user-side energy storage in cloud energy storage mode: the economic operation of user-side energy storage in cloud energy storage mode can reduce operational costs, improve energy storage efficiency, and achieve a win–win situation for sustainable energy development and user economic benefits. Ron D. Rappaport et al. studied the economic feasibility of aggregating distributed energy storage dispatch in the UK electricity market environment^18^. Hendrik Broering et al. researched the advantages of distributed energy storage aggregation dispatch in the German electricity market environment^19^. They proposed an economic evaluation model for cloud energy storage considering current grid electricity prices and other parameters. Pratyush Chakraborty and Li Xianshan et al. introduced an optimization model with the goal of minimizing shared energy storage costs, achieving optimal objectives for shared energy storage charging and discharging, as well as capacity allocation^20,21^. Li Jianlin et al. studied the operational system of three entities, including shared energy storage stations, operators and users^22^. They used a Nash bargaining model for service pricing, considering the initial investment costs of each shared energy storage station for profit redistribution. Zhang Wei et al. proposed a cloud energy storage leasing mechanism, introduced a robust optimization model, and studied the optimal optimization strategy of the leasing mechanism and wind storage coordination^23^. Wang Zhimin and Li Lin et al. achieved the goal of reducing the operating costs of user groups by coordinating the charging and discharging power of shared energy storage^24,25^.

In summary, scholars at home and abroad have studied and explored the cloud energy storage business to some extent in terms of operation mode and scheduling strategy. However, most of them have studied the planning and scheduling of energy storage resources and shared energy storage configuration^26,27^. Few scholars specialize in the coordinated scheduling model of user-side distributed energy storage devices under cloud energy storage mode, including the business model and service mechanism of system operation. The shortcomings of the existing research are summarized as follows^28,29^. (1) Most current user-side small devices energy storage are decentralized, with small capacity, low technology level and no interconnection, not to mention joint dispatch. (2) There is a lack of a proper dispatching mechanism and an intelligent communication system between distribution networks-scale energy storage devices. Consequently, these devices cannot be economically dispatched based on the load demand networks.

Firstly, considering the aforementioned shortcomings this study establishes the architecture and service mechanism of a cloud energy storage system. Secondly, based on the demand and supply of small energy storage devices on the user side and the distribution network, a day-ahead power scheduling model and matching strategy are constructed to ensure optimal overall benefits of the system. The proposed model in this study takes into account predicting electricity supply/demand from cloud storage. Additionally, it adopts a user bid matching trading mode to ensure optimal overall benefit of the system. Finally, taking an integrated energy smart park as an example, the reasonableness and effectiveness of the proposed mechanism are verified by an example analysis. The research content of this paper is conducive to the aggregation of user-side scattered energy storage devices, the formation of scale effect, and ensure the coordinated scheduling of cloud energy storage, to maximize system benefits.

## Cloud energy storage service system architecture and operation mode

### System architecture

**Figure 1:**
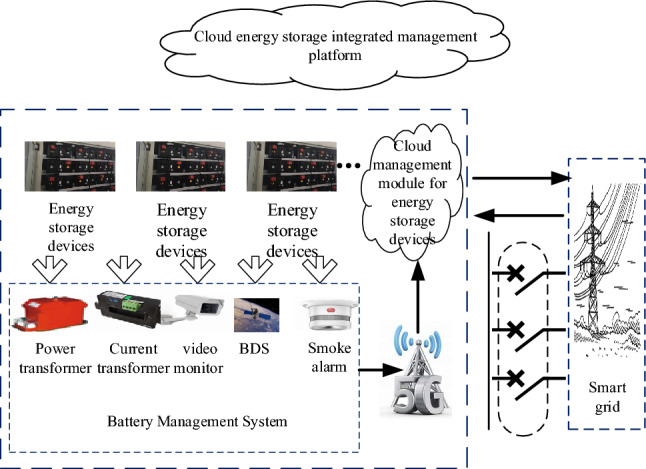
The overall framework of cloud energy storage integrated management services.

Cloud energy storage refers to an energy storage type that utilizes cloud computing technology to connect and manage energy storage systems through the Internet. It involves integrating energy storage devices with intelligent data analysis and control systems, enabling remote monitoring and management of storage systems. The goal of cloud energy storage is to improve energy utilization efficiency and flexibility. The basic principle is connecting distributed energy to cloud servers. The cloud energy storage system takes small user-side energy storage devices as the main body and fully considers the integration of new energy large-scale grid connection and source-grid-load-storage. The cloud energy storage integrated service platform is a cloud energy storage ecosystem built based on battery energy storage, combined with advanced technologies such as the Internet of Things, 5G, big data, cloud services and blockchain. The main body of which includes three categories: the cloud energy storage service provider, small user-side energy storage devices participating in cloud sharing, and distribution networks. The relationship between the participating subjects of the cloud energy storage service is centered on the cloud energy storage service provider. Distribution networks and user-side small energy storage devices are the target customer groups of the service business. Based on the cloud energy storage service system platform, the cloud energy storage builds a valuable information channel between small energy storage devices and distribution networks to realize flexible dispatching of energy storage. Under the information decision of the cloud energy storage service provider, small energy storage devices and distribution networks realize the electric energy trading between each subject through the cloud platform. The technical architecture is roughly divided into three layers. Build a cloud energy storage integrated management service system to support the management and coordinated dispatch of energy storage devices clusters to distribution networks. Build a battery management system for small energy storage devices to obtain the parameters of the battery modules and monitor their charging and discharging conditions and operation status. Build a cluster management system of small energy storage devices on the customer side, collect the operation data of small energy storage devices and upload them to the cloud in real time. Through the cloud energy storage management system, the joint scheduling of multiple energy storage devices is realized, and the optimal allocation of electric energy is realized. The overall framework of cloud energy storage integrated management services is shown in Fig. 1.

### Operation mode

**Figure 2:**
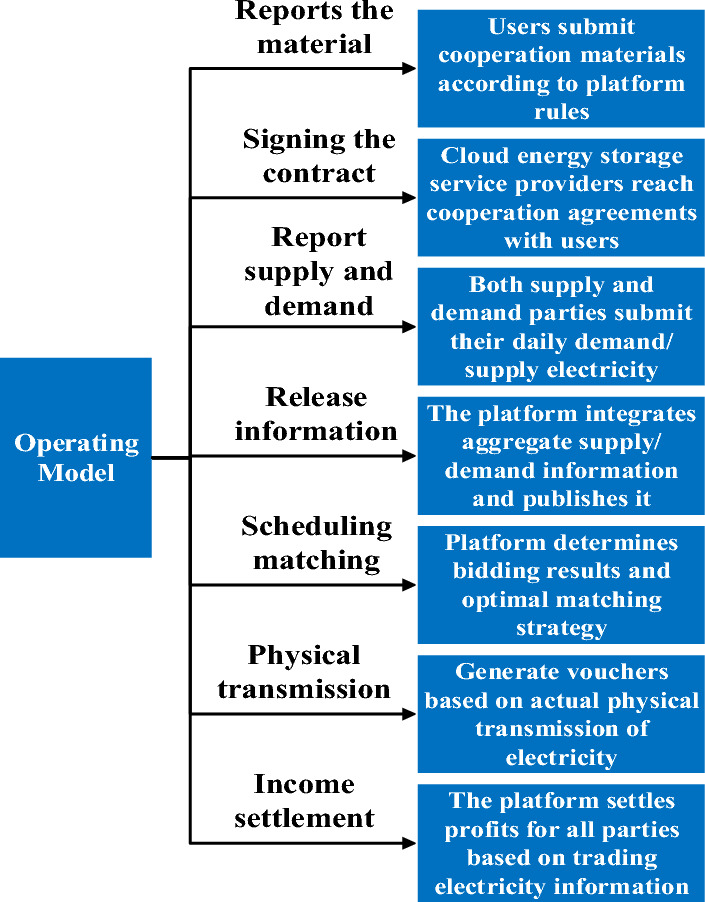
Cloud Energy Storage Service Mechanism Business Process.

The main sources of customers for the cloud energy storage operators are energy storage users who expect to benefit from the peak-to-valley load differential and distribution networks that want to purchase power from the storage devices. During the period when the battery storage device is in surplus, the storage device reports the power that can participate in the dispatch to the cloud energy storage service platform. The cloud energy storage service provider converges the power that each small storage device can participate in the dispatch according to the aggregated power. And according to the optimal scheduling mode, the scheduling plan of each energy storage plant is formulated to supply power to the demand side of distribution networks. The specific operation mode of the cloud energy storage service platform is shown in Fig. 2.

The advantage of the cloud energy storage model is that it provides an information bridge for both energy storage devices and the distribution grid without breaking industry barriers and improves the efficiency of energy exchange. The perfect management mechanism of the cloud energy storage platform provides an orderly and stable scheduling platform for user-side energy storage devices to connect to distribution networks. The reasonable scheduling matching strategy of the cloud energy storage platform can adequately schedule the energy storage devices, which is conducive to reducing the cost per unit of energy storage and improving the income of the storage side. The traditional way of direct connection of multiple energy storage devices to distribution networks is just an integrated use of energy storage resources. It cannot solve the problem of high energy storage costs. Since the traditional way does not have a third-party platform to assist in dispatching, it will lead to instability problems in its overall dispatching.

Firstly, the cloud energy storage service provider makes the platform operation rules. The rules include the platform, small energy storage devices and distribution networks three subjects in the platform have the power, transaction rules, service processes and rules and regulations. Small storage devices and distribution networks need to sign specific contracts if they want to cooperate with the cloud energy storage service provider. Secondly, each subject formally participates in the cloud energy storage platform operation and scheduling. User-side small energy storage devices as well as the power grid need to be submitted to the platform before the day supply/demand power information. The platform side needs to sort out the total supply of power and total demand power information for each time period and release the information. In the bidding and scheduling matching phase, the cloud energy storage platform conducts centralized bidding based on the quotations of small energy storage devices. The platform determines the matching supply and demand sides, the transaction power and the transaction price to achieve the optimal dispatching strategy. On the day of contract execution, the cloud energy storage service provider sends the day-ahead trading plan to the physical transmission side. The physical transmission party controls the charging and discharging to realize the electric energy delivery. Finally, the platform settles the revenue of each party according to the traded electricity.

The goal is to minimize the total system cost during the operation and dispatch of the cloud energy storage service provider. The total system cost is shown in Eq. (1)

1 $$\begin{array}{c}{minC}_{ALL}^{T}=\sum_{t=1}^{24}({C}_{W}\left(t\right)+{C}_{Z}(t)+{C}_{D}(t)) \end{array}$$

where ${C}_{ALL}^{T}$ represents the total cost of operating the control process in one day; $t$ is the unit time; ${C}_{W}(t)$ represents the operation and maintenance cost of the cloud energy storage platform; ${C}_{Z}(t)$ represents the cost of purchasing electricity from distribution networks for small energy storage devices; ${C}_{D}(t)$ represents the cost of purchasing electricity from distribution networks to small energy storage devices.

The specific functions of the above costs are shown below.

1. The operation and maintenance cost of the cloud energy storage platform is shown in Eq. (2).
  2 $$\begin{array}{c}{C}_{W}(t)={C}_{m}{P}_{CSS,t} \end{array}$$
  where ${C}_{m,i}$ represents the unit output operation and maintenance cost coefficient of the *i* − th small energy storage device; ${P}_{CES,t}$ represents the total amount of electricity transactions completed on the cloud energy storage platform during period *t*.
2. The cost of electricity purchased by distribution networks during peak load periods is shown in Eq. (3).
  3 $$\begin{array}{c}{C}_{D}(t)={C}_{Pd}(t){P}_{gpe}(t) \end{array}$$
  where ${C}_{Pd}(t)$ represents the purchase price during period *t*; ${P}_{gpe}(t)$ represents the purchase of power at time *t*.
3. The cost of a small energy storage device to purchase power during low load periods is shown in Eq. (4).
  4 $$\begin{array}{c}{C}_{Z}(t)={C}_{Pz}(t){P}_{dpe}(t) \end{array}$$
  where: ${C}_{Pz}(t)$ represents the purchase price during time *t*; ${P}_{dpe}(t)$ represents the power purchased during time *t*.

### Current power dispatching basis

The forecasting and planning of future electricity demand and supply is known as day-ahead scheduling. The information provided by distribution networks and small energy storage devices should be integrated with the support of cloud energy storage resources. The cloud platform publishes the day-ahead electric energy dispatch plan and distributes and announces it to the participating entities. This information is used as the information condition for the subsequent bidding matching stage. It can be specifically divided into distribution networks power purchase to small energy storage device total electric energy dispatch basis. Firstly, the demand function of the small energy storage device, which is the premise for the rest of the function formulation. The demand function of the energy storage device is shown in Eq. (5).

5 $$ {f}_{ESS}= \left\{\sum_{i=1}^{n}{P}_{dpe,t}\right\} $$

where ${f}_{ESS}$ represents the demand function of small energy storage device; ${P}_{dpe,t}$ represents the total power that small energy storage devices need to purchase during the trough period at time *t*.

Secondly, the demand function of distribution networks, must meet the demand distribution networks demand for each moment for the purpose. The demand function of distribution networks is shown in Eq. (6).

6 $$ {f}_{prid}= \left\{\sum_{i=1}^{n}{P}_{gpe,t} \right\} $$

where ${f}_{prid}$ represents the demand function of the distribution network; ${P}_{gpe,t}$ represents the total power that needs to be purchased by the distribution network at time* t* during the peak period.

Finally, the scheduling function of the cloud energy storage platform is shown in Eq. (7).

7 $$ {f}_{cfh}= \left\{\sum_{i=1}^{n}{d}_{prid,z},\sum_{i=1}^{n}{P}_{gpe,t} \right\} $$

where ${f}_{cfh}$ represents the scheduling function of the cloud energy storage platform; ${d}_{prid,z}$ represents the distance from the small energy storage device to the load side.

The following is the total power dispatch function for the whole system as shown in Eqs. (8) and (9).

8 $$ {f}_{z}=\left\{\sum_{i=1}^{n}{P}_{sup,t},\sum_{i=1}^{n}{P}_{dpe,t},\sum_{i=1}^{n}{P}_{gpe,t},\sum_{i=1}^{n}{P}_{l,t}\right\} $$

9 $$ \sum_{i=1}^{n}{P}_{sup,t}=\sum_{i=1}^{n}({P}_{gpe,t}-{P}_{dpe,t})+\mu \left(\sum_{i=1}^{n}{P}_{s-1,t}-{E}_{SOC,max}\right) $$

where ${f}_{z}$ represents the scheduling function of the total electric energy of the whole system; ${P}_{sup,t}$ represents the total potential power supply of the small energy storage device *i* at time *t*. ${P}_{l,t}$ represents the online clearing power of small energy storage device *i* at time *t*; ${P}_{s-1,t}$ represents the energy storage capacity that the small energy storage device *i* can supply at time *t*-1; ${E}_{SOC,max}$ represents the highest electricity level.

### Constraints

1. System power balance constraint
  Considering the role of the cloud storage operator, the power balance constraint should be maintained for the whole system. The system power balance constraint is shown in Eq. (10).
  10 $$\begin{array}{c}{P}_{LOA,t}={P}_{ESS,t}={P}_{CES,t} \end{array}$$
  where ${P}_{LOA,t}$ represents the peak load demand of distribution networks during time *t*; ${P}_{ESS,t}$ represents the total output of small energy storage devices participating in cloud platform scheduling during time *t*.
2. Upper and lower power limit constraints for small energy storage devices
  Based on the study based on small battery energy storage devices, the maximum and minimum power limits are required to protect the battery life. The energy storage device power constraint is shown in Eq. (11).
  11 $$\begin{array}{c}{E}_{SOC,min}\le {E}_{i,t}\le {E}_{SOC,\mathrm{max}} \end{array}$$
  where ${E}_{SOC,min}$ represents the minimum charge; ${E}_{i,t}$ represents the remaining power.
3. The power constraint for charging and discharging small energy storage devices is shown in Eq. (12).
  12 $$\begin{array}{c}{P}_{c,max}\le {P}_{i,t}\le {P}_{f,max} \end{array}$$
  where ${P}_{c,max}$ represents the maximum charging power of a small energy storage device; ${P}_{f,max}$ represents the maximum discharge power of a small energy storage device; ${P}_{i,t}$ represents the real-time charging power of a small energy storage device.
4. The power dispatch constraints for safe operation of distribution networks are shown in Eqs. (13) and (14).
  13 $$\begin{array}{c}{P}_{LOA,t}\le {P}_{safe,t} \end{array}$$
  14 $$\begin{array}{c}{P}_{dpe,t}\le {P}_{safe,t} \end{array}$$
  where ${P}_{safe,t}$ represents the maximum power that distribution networks can carry during safe operation.

## Cloud energy storage scheduling matching strategy

The cloud energy storage service platform will screen, process and integrate the collected information to generate a variety of transaction matching strategies. Subsequently, the electric energy generated by user-side small energy storage devices will be uniformly scheduled to the distribution network, following the cloud energy storage scheduling matching strategy. The specific steps are as follows:

Step 1: The energy storage service provider collects the power and capacity monitoring data of the contracted small energy storage devices and the distance of the load. The service provider publishes information on the forecasted leasable energy storage and power purchase for the whole network and information on the power of small energy storage devices that can be leased and requested for energy storage in the T_N_ cycle. Information cloud record is recorded by the cloud energy storage information management module to record real-time small-scale energy storage devices leasing and power purchase information as well as the distribution network in the peak period to small-scale energy storage devices power purchase data. The information recorded in the cloud is shown in Eq. (15).

15 $$\begin{array}{c}{U}_{ESA,i}=\{{Q}_{iZL,T},{Q}_{iZM,T}, {p}_{iZL,T},{p}_{iZM,T}\} \end{array}$$

where ${U}_{ESA,i}$ represents the collection of various information and data released by the cloud energy storage service provider to the entire networks; ${Q}_{iZL,T}$ represents the amount of electricity that a small energy storage device *i* can lease for energy storage during the T_N_ cycle; ${Q}_{iZM,T}$ represents the amount of electricity that a small energy storage device *i* needs to purchase for energy storage during the T_N_ cycle; ${p}_{iZL,T}$ represents the price of renting a unit of energy storage capacity for a small energy storage device *i* during the T_N_ cycle; ${p}_{iZM,T}$ represents the price at which a small energy storage device *i* seeks to purchase a unit of energy storage during the T_N_ cycle.

${Q}_{iZL,T}$ is calculated as shown in Eq. (16).

16 $$\begin{array}{c}{Q}_{iZL,T}={Q}_{iZrem,{T}_{n-1}}+{Q}_{iZsel,{T}_{n-1}} \end{array}$$

where ${Q}_{iZrem,{T}_{n-1}}$ represents the actual remaining energy storage capacity of the small energy storage device *i* during the T_N−1_ cycle; ${Q}_{iZsel,{T}_{n-1}}$ represents the energy storage capacity that a small energy storage device requires during the T_N-1_ cycle.

${Q}_{iZM,T}$ is calculated as shown in Eq. (17).

17 $$\begin{array}{c}{Q}_{iZM,T}={K}_{ESA}{Q}_{iZL,T} \end{array}$$

where ${K}_{ESA}$ represents the prediction coefficient of the cloud energy storage service provider *i* obtained by analyzing multiple transaction data of the same period of time.

Step 2: The information reported by both parties is integrated according to the cloud energy storage platform and filtered through smart contracts. The platform issues the optimal power purchase strategy for distribution networks. The distribution network confirms the order and the cooperation between the two parties is reached. The platform service provider records each transaction in the form of cloud storage for subsequent data processing.

At this stage, the cloud energy storage service platform, to determine the matching information between supply and demand. According to the power purchase plan of distribution networks, the supply and demand matching queue is formed by the price quoted by the users of small energy storage devices. The supply price is in the order of “lowest to highest”, and the small energy storage device with the lower price is at the front of the queue. The platform calculates the total power of the supply orders at the front of the queue. The transaction price of each order is the smaller value of the quotation for each time period. The matching strategy function is shown in Eq. (18).

18 $$\begin{array}{l}\left\{\begin{array}{c}{p}_{dhts}^{h}=min\sum_{i=1}^{n}{p}_{shts,i}^{h}\\ {C}_{z}^{h}=\sum_{i=1}^{n}{Q}_{dhts,i}^{h}{ p}_{y,i}^{h}\\ {p}_{dhts}^{h}\le {p}_{iZL,T}\end{array}\right. \end{array}$$

where ${p}_{dhts}^{h}$ represents the transaction price of the unit of electric energy of the bidding network; ${p}_{shts,i}^{h}$ represents the quotation of each small energy storage device; ${C}_{z}^{h}$ represents the total power purchase cost of the distribution network in this round; ${Q}_{dhts,i}^{h}$ represents the transaction capacity of small energy storage device *i*; ${p}_{y,i}^{h}$ represents the quotation of electric energy of this round of small energy storage device *i*.

Subjects of supply and demand successfully matched in this round of quotations exit the queue. Subjects that are not successfully matched continue to adjust their offers to post information on the platform to participate in the next round of bidding. The supply and demand sides match until all demand is met by the N-th iteration. To sum up, the energy storage devices are subject to multiple rounds of bidding starting from moment *t*. Eventually the platform determines the day-ahead electric energy trading bidding results and the optimal matching strategy.

Step 3: The platform sends the orders contracted by the supply and demand sides to the physical transmission service provider for electricity transmission, and the transmission cost is borne by the demand side. After the electricity transmission is completed, the actual electricity transmitted is reported to the cloud energy storage platform and the electricity trading voucher is issued. It is shown in Eq. (19).

19 $$\begin{array}{c}{U}_{ESA,i}=\{{Q}_{iSL,T}, {p}_{iSL,T}\} \end{array}$$

where ${U}_{ESA,i}$ represents the set of power actually transmitted by the system; ${Q}_{iSL,T}$ represents the actual energy storage capacity sold by the small energy storage device *i* during the T cycle; ${p}_{iSL,T}$ represents the price per unit of energy storage sold by the small energy storage device *i* during the T cycle.

Step 4: According to the information of power traded on the cloud energy storage platform, settle the revenue of each party as shown in Eq. (20).

20 $$\begin{array}{c}{\varphi }_{final}\left(Y\right)=k{P}_{CES,t}-{C}_{W}(t) \end{array}$$

where ${\varphi }_{final}\left(Y\right)$ represents the revenue of the cloud energy storage platforms; *k* represents the price for completing the extraction of unit energy storage platform.

The small energy storage device gain is shown in Eq. (21).

21 $$\begin{array}{c}{\varphi }_{final}\left(ES\right)=\sum_{t=1}^{24}{Q}_{iSL,t}{p}_{iSL,t}-\sum_{t=1}^{24}{Q}_{iZM,t},{p}_{iZM,t}-\varepsilon ({Q}_{iSL,t}+{Q}_{iZM,t}) \end{array}$$

where ${\varphi }_{final}\left(ES\right)$ represents the benefit of small energy storage devices; ${Q}_{iSL,t}$ represents the electricity sold during the peak load period of small energy storage devices; ${p}_{iSL,t}$ represents the price per unit of electricity sold; ${Q}_{iZM,t}$ represents the amount of electricity purchased by small energy storage devices during low load periods; ${p}_{iZM,t}$ represents the unit electricity purchase price. $\varepsilon$ represents the operation and maintenance cost factor per unit power of small energy storage input or output.

## Example and analysis

### Example parameter settings

The study verifies the feasibility and effectiveness of the power coordination and optimization dispatch mechanism of the distribution network under the cloud energy storage mode. Five small energy storage devices on the user side of an integrated energy smart park are selected as the object of calculation. The distributed device capacities of small energy storage devices 1, 2, 3, 4 and 5 are shown in Table 1. The basic tariff is based on the transformer capacity and the local capacity tariff is used as the basic capacity tariff. In addition, the energy storage battery type is taken as lithium iron phosphate battery.

**Table 1:** Energy storage devices system parameters.

Energy storing devices	System parameters of energy storage devices		
	Configuration capacity (KWh)	SOC_max_	SOC_min_
1	300	0.9	0.1
2	100	0.9	0.1
3	350	0.9	0.1
4	150	0.9	0.1
5	200	0.9	0.1

## Result

In this study takes the time period from 6 p.m. to 7 p.m. as an example to analyze how the cloud energy storage platform dispatches the five energy storage devices in the scenario of the energy smart park. The aim is to assist the park's distribution network in adjusting its frequency and managing peak demand during this time frame. The bidding matching process between the two trading parties on the cloud energy storage platform is resolved using Eq. (18). The energy storage device reported to the cloud energy storage platform from 6 p.m. to 7 p.m. can supply electricity. The electrical energy supplied by the energy storage device is shown in Table 2. This time, the distribution network's power demand is 675 kWh. The details of the online bidding process for energy storage devices are presented in Table 3.

**Table 2:** The electrical energy provided by an energy storage device.

Energy storage devices	1	2	3	4	5
Electric quantity (kW h)	265	80	315	125	175

**Table 3:** Bidding process.

Energy storage devices	Prices for different bidding rounds (CNY/kWh)				
	First round	Second round	Third round	Fourth round	Fifth round
1	0.4873	–	–	–	–
2	0.4939	0.4763	0.4563	0.4421	–
3	0.4998	0.4635	0.4611	0.4523	0.4507
4	0.5021	0.4546	–	–	–
5	0.5034	0.4628	0.4539	–	–

As can be seen from Table 3 of this study, energy storage device 1 had the lowest bid in the first round of bidding and successfully matched with the distribution network. However, during this period, the power demand and supply of the distribution network remained unbalanced. Consequently, the distribution network had to engage with the lowest bidder again in the subsequent round of bidding. This bidding process continued until the electricity demand of the distribution network was met. The specific transaction matching results of the supply and demand parties during this period are shown in Table 4.

**Table 4:** Matching and scheduling results.

Bidding rounds	Matching result between supply and demand	Electric quantity (kW h)	Transaction price (CNY/(kW h))
1	Energy storage device 1-distribution network	265	0.4873
2	Energy storage device 4-distribution network	125	0.4546
3	Energy storage device 5-distribution network	175	0.4539
4	Energy storage device 2-distribution network	80	0.4421
5	Energy storage device 3-distribution network	30	0.4507

**Figure 3:**
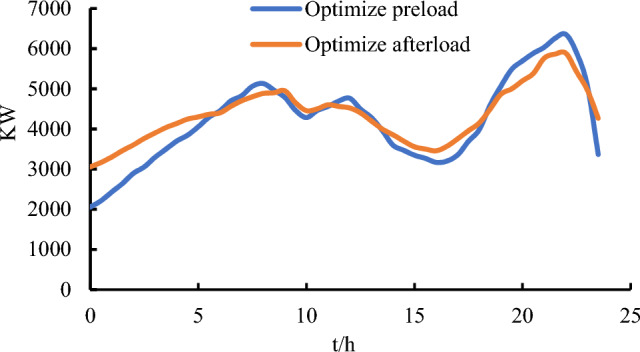
Load optimization results.

In this study, demand-side load data were collected before and after the participation of cloud energy storage in power grid FM service, and the comparison results are shown in Fig. 3. The load curve is smoother after optimization compared to before. After load optimization, the small energy storage device purchases power from the distribution network to supply the storage device itself during the low load period, increasing the demand-side load during the low period. The small storage device sells power to the distribution network during peak hours to regulate the peak demand-side load and reduce the load during peak periods. At the same time, users of the cloud energy storage can choose to discharge when electricity prices are high and charge when prices are low. The supplier realizes cloud energy storage scheduling as well as the purpose of optimal economic returns on this basis.

**Figure 4:**
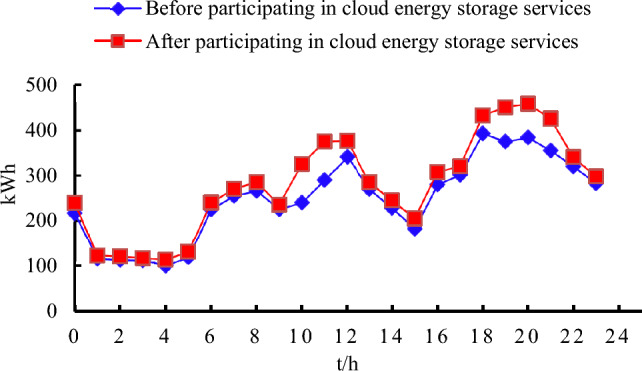
Comparison of electricity sold by small energy storage devices 1–5 before and after participating in the service.

In this study, the electricity sales data of five user-side small energy storage devices were collected for a period of 24 h both before and after their involvement in cloud energy storage services. As depicted in Fig. 4, there is a significant increase in the amount of electricity sold by these devices after participating in energy storage services, particularly during periods of peak electricity consumption. Furthermore, the total daily sales of these energy storage devices experienced a growth rate of 19.06%. This reflects positively that, under the condition of unchanged demand on the load side, the overall utilization rate of small energy storage devices has been improved due to resource optimization and scheduling by the cloud energy storage service provider. Small energy storage devices purchase electricity during the low load period of the distribution network, ensuring the economic benefits of the energy storage party.

**Figure 5:**
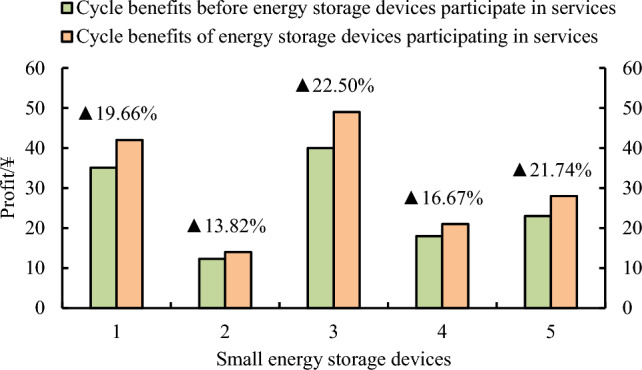
Comparison of benefits of small energy storage devices before and after participating in cloud services.

The income from the energy storage device determined by Eq. (21). Furthermore, the study evaluates the benefits of cooperation between small-scale energy storage devices on the user side and cloud energy storage service providers before and after. The ratio of leased capacity to actual storage capacity of the storage device at full power is 0.9. The small storage device is scheduled to complete the transaction with the distribution network through the cloud storage platform. As shown in Fig. 5, the revenue of distributed small energy storage devices 1–5 after participating in cloud energy storage services increased by 19.66%, 13.82%, 22.50%, 16.67% and 21.74%, respectively, compared with that before participating in the service. In various scenarios, according to the optimal scheduling mode of the cloud energy storage, the utilization rate of customer-side energy storage devices can be fully improved. It makes the energy storage device a container for the low purchase and high sale of electric energy and obtains the economic benefits of the transaction. As can be seen from the figure, comparing the returns of five types of small energy storage devices after participating in the cloud service can be seen. In the unit cycle, the cycle gain after the small energy storage device participates in the service is greater than the cycle gain before the small energy storage device participates in the cloud service.

It can be seen that the cloud energy storage service mechanism can maximize the efficiency of dispatching individual customer-side small energy storage devices to achieve maximum resource utilization. Small energy storage devices sell electricity to the distribution network during peak periods and purchase electricity from the distribution network during low periods. Using the difference between peak and valley electricity prices can maximize economic benefits and reduce energy costs. The cloud energy storage service platform fully exploits the value of decentralized energy storage resources to participate in grid load regulation.

In summary, the optimal dispatching methods proposed in the study can all help to cut peaks and fill valleys in the distribution network while providing correct and reasonable dispatching of small energy storage devices on the customer side. The addition of the cloud energy storage platform makes up for the situation that small energy storage devices in the distribution network cannot be dispatched adequately. The cloud energy storage service enables a significant increase in the utilization of energy in storage devices. The cloud energy storage can also make full use of the energy storage devices through reasonable charging and discharging strategies so that users can gain benefits. The cloud energy storage service can smooth the load curve and reduce the load peak-to-valley difference in the distribution network. In the future, the cloud energy storage platform has broad applications in optimizing the dispatch of small devices on the user side.

## Discussion

### Previous studies and this study

The existing research on cloud energy storage mainly focuses on resource planning and scheduling and economic optimal allocation, and there are few researches on user-side distributed energy storage. Therefore, distributed energy storage has not yet formed scale benefits, and the scheduling mechanism is not perfect^30,31^. However, this study focuses on the optimal scheduling of user-side small energy storage devices, which makes up for the shortcomings of previous studies. The specific differences are as follows:

1. User-side small energy storage participates in the optimization and scheduling of the cloud energy storage service platform, which can aggregate dispersed energy storage devices. It realizes the interconnection between the energy storage devices, forms a good scale effect, and fully improves the utilization rate of small energy storage equipment resources. At the same time, this mode can reduce the cost of electricity for users equipped with energy storage devices, and indirectly improve the economic benefits of users.
2. The optimal scheduling mechanism in this study can predict the electric energy of both sides of the transaction. In this study, the supply side and the demand side are coordinated and planned by adopting the bidding matching trading mode. This method not only reduces the waste of resources such as wind abandonment and light abandonment, but also promotes the further improvement of the shared energy storage scheduling mechanism, and provides research support for the future aggregate scheduling of small-scale energy storage equipment at the user side.

### Limitations and future research lines

This study does not consider the corresponding security mechanism and privacy protection measures. At the same time, this paper only considers the general regional characteristics, and does not consider the complex situation of special regions.

In the future, it is necessary to study the data security and privacy protection technology of cloud energy storage platform to ensure the security of energy data and user privacy involved in the user-side energy storage system. This includes research on encryption algorithms, authentication mechanisms, distributed data management, etc., in order to deal with the growing challenges of network security. In addition, future research should consider a more comprehensive combination of distributed energy storage resources and centralized energy storage, develop more diversified forms of cloud energy storage services for different types of users, and build an energy storage trading market based on full competition.

## Conclusion

In this study proposes a coordinated and optimized scheduling mechanism for user-side energy storage based on the concept energy storage. The main conclusions are as follows:

1. The system architecture and operation mode of cloud energy storage proposed based on the characteristics of user-side distributed energy storage have laid the foundation for the commercialization of cloud energy storage.
2. Based on the day-before optimal scheduling model and forecast information, the cloud energy storage service provider formulates a cluster scheduling matching strategy for energy storage devices, which ensures the economic benefits of users, improves the consumption space of new energy, and promotes the peaking and valley filling of the power grid.
3. Based on electric energy trading bidding and matching strategy, it improves market competitiveness and helps to stimulate user autonomy and transaction efficiency.

The user-side energy storage coordination and optimization scheduling mechanism proposed in this study under cloud energy storage mode helps the power grid optimize the load peak-valley difference. This method also fully improves the utilization rate and income of user-side small energy storage device resources, maximizes the utilization value of decentralized energy storage resources, and promotes the progress of the new generation of power grid peak regulation and frequency regulation business.

## Data Availability

The data that support the findings of this study are available from [Power companies in China] but restrictions apply to the availability of these data, which were used under license for the current study, and so are not public available. Data are however available from the authors upon reasonable request and with permission of [Powel companies in China]. If anyone needs to obtain data, please contact Huidong Wang. E-mail: wangyuqing990701@163.com.

## References

[1] *Actively and Steadily Promote Carbon Neutral Carbon Peaks*. http://www.gov.cn/yaowen/2023-04/06/content_5750183.htm.

[2] *The “Two Alternatives” Deep Promotion Faces Multiple Difficulties*. http://ccnews.people.com.cn/n1/2018/1010/c141677-30331917.html.

[3] Wu S-J, Liu J-K, Zhou Q, Wang C-G, Chen Z (2019). Optimal economic scheduling for multi-microgrid system with combined cooling, heating and power considering service of energy storage station. Power Syst. Autom..

[4] Zhao X-Q, Qin L-J, Duan H (2020). Distributed energy storage optimal scheduling of distribution networks based on aggregation effect. Power Capacitors React. Power Compens..

[5] Zhu J-Z (2023). Review of demand-side energy sharing and collective self-consumption schemes in future power systems. iEnergy..

[6] Dai R, Esmaeilbeigi R, Charkhgard H (2021). The utilization of shared energy storage in energy systems: A comprehensive review. IEEE Trans. Smart Grid.

[7] Byrne R, Nguyen T, Copp D, Chalamala B, Gyuk I (2017). Energy management and optimization methods for grid energy storage systems. IEEE Access.

[8] Liu J-K, Zhang N, Kang C-Q, Kirschen D, Xia Q (2017). Decision-making models for the participants in cloud energy storage. IEEE Trans. Smart Grid.

[9] Li X-Y, Chen G, Dong Z-Y (2020). Co-optimisation model for the long-term design and decision making in community level cloud energy storage system. IET Renew. Power Gener..

[10] Zhou R-J (2020). Industrial and commercial electricity cost reduction optimization plan using cloud energy storage service. South. Power Syst.Technol..

[11] Li X-S, Chen A-B, Cheng S, Chen M-R (2021). Multi-agent coordination and optimal dispatch of microgrid with CES based on ecological game. Electric Power.

[12] Appino R, Hagenmeyer V, Faulwasser T (2021). Towards optimality preserving aggregation for scheduling distributed energy resources. IEEE Trans. Control Netw. Syst..

[13] Zhang N (2021). Aggregating distributed energy storage: Cloud-based flexibility services from China. IEEE Power Energ. Mag..

[14] Ma Y-C (2022). Research on peer-to-peer transaction strategy of cloud energy storage based on semi-distributed structured topology. Proc. CSEE.

[15] Wu S-J, Li QZ, Liu J-K, Zhou Q, Wang C-G (2021). Bi-level optimal configuration for combined cooling heating and power multi-microgrids based on energy storage station service. Power Syst. Technol..

[16] Ibrahim L (2022). Coordinated frequency control of an energy storage system with a generator for frequency regulation in a power plant. Sustainability.

[17] Guo Y-Z (2020). Comprehensive optimization configuration of electric and thermal cloud energy storage in regional integrated energy system. Power Syst. Technol..

[18] Rappaport RD, Miles J (2017). Cloud energy storage for grid scale applications in the UK. Energy Policy..

[19] Broering H, Madlener R (2017). Simulation and evaluation of the economic merit of cloud energy storage for prosumers: The case of Germany. Energy Proc..

[20] Chakraborty P, Baeyens E, Poolla K, Khargonekar P, Varaiya P (2018). Sharing storage in a smart grid: A coalitional game approach. IEEE Trans. Smart Grid.

[21] Li X-S, Xie S-J, Fang Z-J, Li F, Cheng S (2021). Optimal configuration of shared energy storage for multi-microgrid and its cost allocation. Electric Power Autom. Equip..

[22] Li J-L (2022). Operation mode optimization and economic benefit analysis of demand-side shared energy storage. Power System Technol..

[23] Zhang W, Lu X (2022). Sharing and self-operating multi-mode trading model of energy storage aggregators with peer-to-peer trade. Autom. Electric Power Syst..

[24] Wang Z-M, Gu C-H, Li F-R, Bale P, Sun H-B (2013). Active demand response using shared energy storage for household energy management. IEEE Trans. Smart Grid..

[25] Li L (2020). Optimal economic scheduling of industrial customers on the basis of sharing energy-storage station. Electric Power Construct..

[26] Nikoobakht A (2019). Assessing increased flexibility of energy storage and demand response to accommodate a high penetration of renewable energy sources. IEEE Trans. Sustain. Energy.

[27] Erdiwansya H (2021). A critical review of the integration of renewable energy sources with various technologies. Protect. Control Mod. Power Syst..

[28] Yuan X-D, Fei J-T, Hu B, Zhang Y-W, Ge L (2019). Joint scheduling model of distributed generation, energy storage and flexible load under resource aggregator mode. Power Syst. Protect. Control.

[29] Sun S, Chen L-J, Qiu X-J, Zheng T-W, Mei S-W (2019). A generation-side shared energy storage planning model based on cooperative game. Global Energy Internet..

[30] Li Y-W (2023). Multi-energy cloud energy storage for power systems: Basic concepts and research prospects. Proc. CSEE.

[31] Zhu G-H (2021). Multi-source coordination and optimization of microgrid based on cloud energy storage. Chin. J. Power Sources.

